# Computed Tomographic Anatomy of the Head in Cockatiel (*Nymphicus hollandicus*)

**DOI:** 10.1002/vms3.70234

**Published:** 2025-01-28

**Authors:** Seyedmehran Kazemi, Mehdi Rezaei, Siamak Alizadeh, Mohammadreza Hosseinchi

**Affiliations:** ^1^ Faculty of Veterinary Medicine, Urmia Branch Islamic Azad University Urmia Iran; ^2^ Department of Clinical Sciences, Faculty of Veterinary Medicine, Urmia Branch Islamic Azad University Urmia Iran; ^3^ Department of Clinical Sciences, Faculty of Veterinary Medicine, Naghadeh Branch Islamic Azad University Naghadeh Iran; ^4^ Department of Basic Sciences, Faculty of Veterinary Medicine, Urmia Branch Islamic Azad University Urmia Iran

**Keywords:** anatomy, cockatiel (*Nymphicus hollandicus*), computed tomography, head

## Abstract

**Background:**

Nowadays, computed tomography (CT) scanning is one of the most practical and precise diagnostic imaging methods that can be utilized to evaluate the head in birds.

**Objectives:**

This study aimed to present the normal anatomical data of the head of the cockatiel (*Nymphicus hollandicus*) using the CT method. In this research, the features of this bird's head were investigated in terms of bones, joints, muscles, sinuses and other constituent tissues.

**Methods:**

The current retrospective cross‐sectional study used carcasses of six adult cockatiels (*Nymphicus hollandicus*) (three males and three females) with an average age of 1–3 years and an average weight of 75–110 g. After preparing the CT images, the head of each parrot underwent gross anatomy studies.

**Results:**

Based on the results, reconstructed CT images could identify most structures of the cockatiel (*Nymphicus hollandicus*) head. Parietal, mandible, occiput, maxillary, premaxillary, palatine, pterygoid, quadrate, temporal bones, epithelial membranes, external ear canal and bony labyrinth, ossicles and entoglossal bones, different parts of the infraorbital sinus, brain hemispheres and various parts of the eyeball and conchae of the nasal cavities were examined in CT images. The results related to the CT evaluation and anatomical examination of the cockatiel (*Nymphicus hollandicus*) head demonstrated a high correlation.

**Conclusion:**

The results of this research can be employed as a reference and a suitable atlas for identifying anatomical features, examining different species of the cockatiel (*Nymphicus hollandicus*), teaching anatomy and interpreting CT scan images, as well as performing clinical examinations and treating this type of parrot.

## Graphical abstract

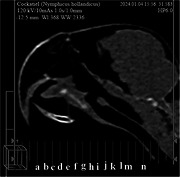

## 1 Introduction

The cockatiel (*Nymphicus hollandicus*) is one of the most popular domestic birds. In many countries, this bird is known as Cockatoo, Weero, Quarrion, Calopsita and Carolina (Fox and Millam 2010; Stevens et al. 2021). The length of this bird is 30–35 cm and it is the smallest species of crested parrots (Liévin‐Bazin et al. 2019). There is a relatively long crest on the head of this bird and a red spot can be seen on its cheeks (Kubiak 2020). The weight of these birds is between 80 and 110 g (de Carvalho et al. 2019). The average life span of these parrots in captivity is 20–30 years, and it has been reported to be more than 40 years in some cases. The heads of parrots are normally large and make up about 15%–20% of the total body weight. The eyes constitute the bulk of the skull and are placed inside a sclerotic pupil. In some parrot species, the lower part of the eye is surrounded by a unique bony arch or suborbital arch (Henley 2018). The rostrum of the parrot is connected to the skull bone by a joint, which gives the rostrum the ability to move upwards. The parrot's tongue is more active and agile due to the entoglossal bones inside the mouth (Benedict et al. 2022). The mandible and maxilla bones of birds are placed inside the upper and lower elements of the beak (Forouzan and Cohen 2021). The mandible contains the nasal cavity, inside which the turbinates, or conchae, are stretched longitudinally. Among the diagnostic imaging techniques, conchae can merely be detected with computed tomography (Langlois et al. 2021). Parrots have distinct sinuses on their faces (Moore et al. 2022). The primary sinus chamber and the infraorbital sinus surround the ventral part of the eyeball and then extend to areas around the eyes and ears through a series of canals. Some of these canals, along with the cervicocephalic air sac, extend to the central concha, the lower jaw and the posterior parts of the neck. Except for the rostral part of the infraorbital sinus, these sinuses can be examined only through CT or magnetic resonance imaging (MRI) (Faux and Logsdon 2022). Parrots lack the prefrontal, postfrontal, temporal and post‐parietal skull bones (Smith‐Paredes et al. 2018). The palatal bones of parrots are small and light. These birds lack teeth and have a large and ossified brain chamber, which leads to weight loss and ease of flight in these parrots (Hollwarth et al. 2023; Carril et al. 2021). Parrots kept at home and able to fly may suffer from head injuries under certain conditions. For instance, the bird may hit the window or land in an inappropriate place, which leads to traumatic injuries. Various imaging techniques can be beneficial in diagnosing these types of damage. Veladiano et al. (2016) examined the natural anatomy of the heads of blue‐and‐yellow macaws (*Ara ararauna*), African grey (*Psittacus erithacus*) and monks (*Myiopsitta monachus*) by CT, labelled different parts of their heads on CT images, and finally introduced the obtained findings as an atlas of the natural head anatomy of these parrots. Likewise, Faillace et al. Santana (2021) investigated the anatomical features of the head of the blue‐fronted Amazon parrot (*Amazona aestiva*) by the CT method. According to this research, some of these features, such as the size and position of the nasal conchae, the infraorbital sinus chamber, the nasopharyngeal duct and the paraglossum, were different in this type of parrot compared to other parrots, which can be used in anatomy analysis. They also reported that the inner ear and its related structures and the paratympanic sinus cannot be well‐examined in normal CT images of this type of parrot. Similarly, Thurber et al. (2015) evaluated the differential diagnosis of parrots' neurological symptoms caused by hydrocephalus syndrome. They concluded that CT is a suitable screening tool for diagnosing hydrocephalus in this type of sick bird. In addition, using potassium iodide contrast medium and CT imaging, Jones et al. (2019) investigated the radioanatomical characteristics of the rock dove, or the common pigeon, especially in the head. They found that CT scanning can be utilized as a preferred method for examining different body tissues of this type of bird, and the images obtained with this method will be a valuable source for clinical applications and educational and research purposes. Using CT, Duymus et al. (2013) compared the head anatomy of white, brown and wild Japanese quails in terms of the head volume, brain volume, parieto‐occipital air space volume and calvarial bone volume and indicated that the head of white quails had the lowest volume values, which was due to genetic differences. Several studies have demonstrated the diagnostic value of CT in the diagnosis of complications and disorders of the head of a parrot. Hébert (2019) confirmed rostroparasphenopalatal luxation in a red‐crowned parakeet (*Cyanoramphus novaezelandiae*) by the CT method, and the bird completely recovered after therapeutic measures. Further, Krautwald‐Junghanns et al. (1998) compared radiology and CT scan techniques in the diagnosis of head diseases in sick parrots and reported the superiority of the CT method in the diagnosis of complications such as fractures of the head bones and identification of hypercalcification or hypocalcification and carcinoma in this area. The investigation of the tomographic features of the head of the rose‐ringed parakeet can be beneficial in identifying anatomical features and evaluating its pathological cases. However, a precise examination of details related to the normal anatomy (morphology and morphometry) of the different parts of this bird's head is necessary. Currently, radioanatomical studies of the head of the cockatiel (*N. hollandicus*) are rare, and there are no detailed reports in this respect. Accordingly, this study aimed to investigate the normal anatomy of the cockatiel (*N. hollandicus*) head by CT using three‐dimensional (3D) modelling. The CT evaluation and anatomical examination of a bird will simultaneously provide valuable findings in this regard. The results of this research can be used as a reference and atlas in identifying anatomical characteristics, investigating different species of cockatiel parakeets, teaching anatomical sciences, interpretation of CT scan images and clinical examinations and treatment of this type of parrot.

## 2 Materials and Methods

### 2.1 Ethical Statement

This work involved the use of procedures that did not differ from established, internationally recognized high standards (best practices) of veterinary clinical care for individual animals. The study was registered under registration code #IR.IAU.URMIA.REC.1403.037 in the Ethical Committee of Islamic Azad University, Urmia.

### 2.2 Study Plan and Birds

The current descriptive cross‐sectional study used carcasses of six adult cockatiels (*N. hollandicus*) (three males and three females) with an average age of 1–3 years and an average weight of 75–110 g, which were well‐fed during their lifetime. The carcasses were obtained from a private breeding centre for cockatiel parrots in Tehran and then frozen and stored at −20^°^C. The parrots, which previously died for various reasons, were used in this study, and the cause of their deaths was unrelated to this study. The maturity of these parrots was confirmed based on factors such as the type of colour of the neck ring, the amount of scales on the feet, the condition of the feathers and the colour of the beak. The sex of the parrot was also determined following a necropsy of the carcass (Webb and Gaston 2000; Vučićević et al. 2016).

### 2.3 Computed Tomography Studies

To prepare CT images, the cockatiel (*N. hollandicus*) was placed on the CT scanner in a sternal recumbency, and the head of the bird was kept facing forward so that its mandible was perpendicular to the gantry. Head scans were performed in the sagittal, transverse and dorsal planes with a thickness and interval of 1 mm. A helical scanner (Toshiba Multi‐slice CT Scanner Asteion Premium 4, Model: TSX‐021B, Japan) was employed for CT. In addition, appropriate windows were selected to examine soft and bone tissues. The technical factors of the CT scanner included gantry rotation time (400 ms), slice thickness (1 mm), reconstruction distance (1 mm), pitch ratio (1), kVp (120), mAs (10), physical detector collimation (32 mm × 0.6 mm), final section collimation (64 mm × 0.6 mm), resolution (512 × 512 pixels), resolution range (0.92 × 0.92), kernel (10 H) and increment (0.5 mm) (Ma et al. 2021; Faillace et al. 2021). Imaging was performed based on the above‐mentioned factors, and the obtained images were saved in DICOM format (Brühschwein et al. 2018).

### 2.4 3D Reconstruction

After saving the obtained images in DICOM format, they were transferred to a computer loaded with 3D modelling software (Onis CT software, Multi‐Modality Workplace: VE 2.5A) and displayed using bone window settings window width (WW): UH 4500 and window level (WL): UH750, according to previous research (Wilhite and Wölfel 2019). Next, these images were analysed with 3D slicer software (Šljivic et al. 2019). Based on our observations, this technique allowed the use of lung (WW: 2336 HU; WL: 368 HU) and bone (WW: 950; WL: 390) windows, thus providing high‐resolution images of the tissues and structures that constitute the head of parrots.

### 2.5 Anatomical Studies

Following the preparation of CT images, the head of each of the frozen parrots was transversely cut with an electric band saw at intervals of 5 mm from the rostral part of the rhamphotheca to the anterior end of the neck. Each prepared slice was cleansed with water and a soft brush and photographed. Visible textures and structures were identified and labelled on these photographs. Further, CT images were matched with these photos and labelled accordingly. Nomina Anatomica Veterinaria was used as the obtained scientific term (International Committee on Veterinary Gross Anatomical Nomenclature 2017).

## 3 Results

**FIGURE 1:**
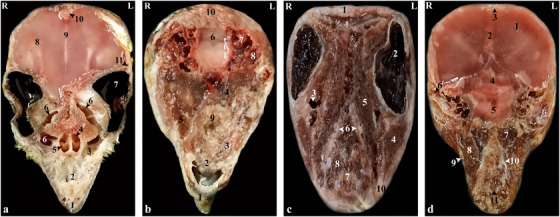
Representative photographs of anatomic cross sections of the adult cockatiel (*Nymphicus hollandicus*) head. (a) Level of the eye, (b) level of the external acoustic meatus in the dorsal plane, (c) level of the rostral border of the orbital fossa and (d) level of the external acoustic meatus in the transverse plane. (a) (1) Rhamphotheca, (2) premaxilla bone, (3) maxilla bone, (4) left nasal cavity, (5) caudal nasal concha, (6) infraorbital sinus, (7) eye, (8) brain hemispheres, (9) falx cerebri, (10) occipital bone, (11) temporal bone. (b) (1) Rhamphotheca, (2) premaxilla bone, (3) palatine bone, (4) ethmomandibularis muscle, (5) pterygoideus muscle, (6) cerebellum, (7) bony labyrinth, (8) external acoustic meatus, (9) caudal nasal concha, (10) occipital bone. (c) (1) Fronto‐parietal bone, (2) eye, (3) infraorbital sinus, (4) pterygoideus muscle, (5) ethmomandibularis muscle, (6) hard palate, (7) eye, (8) caudal nasal concha, (9) lingual process of hyoid bone, (10) tongue, (11) choanal cleft, (12) mandible. (d) (1) Cerebrum, (2) falx cerebri, (3) occipital bone, (4) brain stem, (5) chiasma optic, (6) external acoustic meatus, (7) ethmomandibularis muscle, (8) pterygoideus muscle, (9) mandible, (10) hard palate, (11) lingual process of the hyoid bone. L, left; R, right.

**FIGURE 2:**
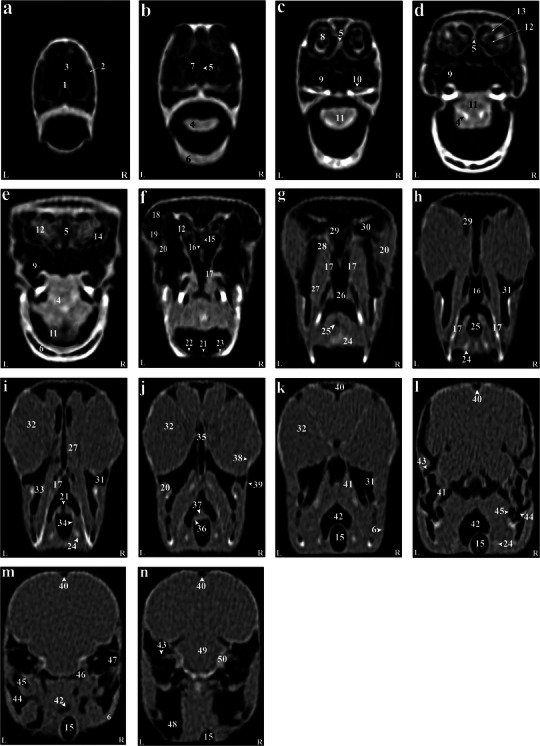
(a–n) Transverse computed tomography reconstruction images in the lateral plane of the normal skull of the cockatiel (*Nymphicus hollandicus*) from the rostrum to the caudal extremity of the nasal cavity. (1) Rostral diverticulum septum, (2) premaxillary bone, (3) rostral diverticulum, (4) paraglossum, (5) bony part of nasal septum, (6) mandible bone (pneumonized), (7) palate bone opening, (8) rostral nasal concha, (9) transverse canal, (10) maxillary process of palatal bone, (11) tongue, (12) middle nasal turbinate, (13) basal layer of middle nasal turbinate, (14) nasal cavity, (15) cartilaginous part of nasal septum, (16) nasopharyngeal airway, (17) lateral border of palatine bone, (18) periorbital process of infraorbital sinus, (19) jugal part of infraorbital sinus, (20) jugal arch, (21) glottis, (22) laryngeal protrusion, (23) arytenoid cartilages, (24) bronchial horn, (25) trachea, (26) choana of palatal bone, (27) ethmomandibular muscle, (28) periorbital part of the infraorbital sinus, (29) caudal nasal turbinate, (30) infraorbital sinus foramen, (31) infraorbital part of the infraorbital sinus, (32) eyeball, (33) epithelial membrane, (34) tracheal cartilage ring, (35) infraorbital septum, (36) cricoid cartilage, (37) procricoid cartilage, (38) scleral ossicles, (39) suborbital arch, (40) frontal bone (pneumonized), (41) pterygoid and quadrate muscles, (42) larynx, (43) zygomatic process of the squamosal bone, (44) quadrate bone (pneumatized), (45) quadrature part of infraorbital sinus, (46) postorbital part of infraorbital sinus, (47) external acoustic meatus, (48) cervicocephalic diverticulum, (49) brain stem, (50) bony labyrinth. L, left; R, right.

**FIGURE 3:**
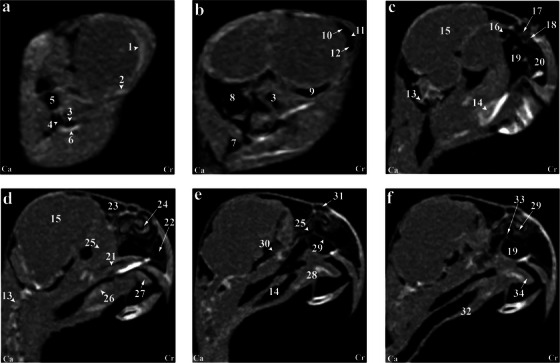
(a–f) Sagittal computed tomography reconstruction images (lateromedial plane) of the normal skull of the cockatiel (*Nymphicus hollandicus*) from the right aspect of the head to the inter‐orbital and nasal septum. (1) Scleral bones, (2) suborbital arch, (3) postorbital part of the infraorbital sinus, (4) quadrate bone (pneumonized), (5) external ear foramen, (6) mandible bone, (7) cervicocephalic diverticulum, (8) occipital bones (pneumonized), (9) infraorbital part of infraorbital sinus, (10) periorbital process, (11) epithelial membrane, (12) jugal portion of infraorbital sinus, (13) cervical vertebrae, (14) trachea, (15) encephalon of the brain, (16) caudal nasal turbinate, (17) middle nasal turbinate, (18) rostral nasal turbinate, (19) transverse canal, (20) premaxillary bone (pneumonized), (21) palate bone (pneumonized), (22) rostral diverticulum, (23) frontal bone (pneumonized), (24) nasal cavity, (25) nasopharyngeal airway, (26) larynx, (27) paraglossum, (28) basihyal, (29) bony part of nasal septum, (30) infraorbital septum, (31) nostril, (32) tracheal rings, (33) cartilaginous part of nasal septum, (34) tongue. Ca, caudal; Cr, cranial.

Based on the results, most structures of the head of the cockatiel (*N. hollandicus*) were detectable by reconstructed CT images. In the 3D images, the head of this parrot was round and compact. The jugal arch and the palatine bone were fused in the remaining parts of the skull, except for the bones of the cranial parts of the face. Small bones of the head, such as ear bones and entoglossal bones inside the mouth, could also be evaluated in these CT images. In this research, it was possible to observe the bony trabeculae in the head of this type of parrot by using the lung window. In addition, parietal and temporal bones, nasal conchae, epithelial membranes, the external ear canal and the bony labyrinth were examined using this filter. Further, with covering tissues, different parts of the infraorbital sinus could be observed using this window. Furthermore, different soft tissue windows were adjusted to identify brain hemispheres, cerebellum, optic nerve, pupil muscles and eye lenses (Figures 1, 2, 3). Based on the findings, the columella ossicle, its external cartilage and the cochlea were undetectable in CT images. The eyeballs of all parrots were complete and bony and located on the skull's lateral side (Figure 1i). The mandible was bony and lacked a distinct symphysis (Figures 1b and 2a). The rostrum was keratinous, large and curved ventrally. Moreover, the operculum could be identified in the dorsal part of the nostrils (the dorsal base of the nose). The occipital, maxillary, premaxillary, mandible, palatine, pterygoid and quadrate bones were pneumonized and had air bubbles. The nasal cavities were separated by a septum. The thickness of this septum slightly increased from the rostral to the caudal side. The caudal third of this septum was cartilaginous, and the middle third and the rostral parts were bony. The ectethmoid, mesethmoid, maxillary and premaxillary bones were involved in the formation of the nasal cavity. The nasal cavity had olfactory, respiratory and vestibular parts. Each nasal cavity had a single duct with caudal, middle and rostral cartilaginous conchae. The rostral concha was C‐shaped and located on the vestibular part of the nasal cavity. The thickness of this concha decreased from the rostral to the caudal direction. The rostral concha contained a basal lamella and was placed on the lateral wall of the nasal cavity. The middle concha was in the form of long ducts that originated from a basal lamella and was located in the upper respiratory tract of the nasal cavity. This lamella also splits into a sinusoidal lamella and a spiral lamella. This spiral lamella extended to the entrance of the nasopharyngeal canal. The caudal concha was small and hollow and was located in the caudal part of the nasal cavity. The nasal and oral cavities were connected through the nasopharyngeal canal (Figures 2c and 3h). The nasopharyngeal duct was connected to the maxilla‐palatal process and the choanal part of the palatine bone from the rostrolateral and caudal sides, respectively. The caudal part of the nasopharyngeal duct was linked to the inter‐orbital septum (Figures 2e and 3f). The oral cavity consisted of palatal, mandible, premaxillary and maxillary bones, as well as their related muscles and tongue. These oral bones, along with the pterygoid, also played a role in the formation of the pharynx (Figure 3c,d). The choana was located in the dorsal part of the pharynx and oral cavity and connected the oral cavity to the nasal cavity (Figure 1g). The strong and large tongue of the cockatiel (*N. hollandicus*) could be identified in the CT images, which was located in the caudal and middle third of the inferior part of the oral cavity (Figure 1c,d). The oral cavity had a hyobranchial apparatus. The base of the tongue was in close contact with the paraglossum and the cranial part of the basihyal. Bishyal processes and uhorial bones were detectable in the trachea's larynx and cranial part. The branchial horn (the caudal part of the hyobranchial apparatus) was located in the inner part of the ramus of the mandible, or the cranial part of the trachea. The caudal third of the branchial horn was related to the mandible masseter muscles. The larynx consisted of a ring‐shaped cricoid cartilage and two pyramid‐shaped arytenoid cartilages. The results of the present study demonstrated that the procricoid cartilage was located in the middle part of the cricoid cartilage and formed the dorsocaudal part of the larynx (Figure 1b–j). The glottis was located in the central part of the larynx and was surrounded by the arytenoid cartilages. Laryngeal mounds (Mons laryngealis) were detectable in cross‐sectional CT images. The place where the cricoid joins the tracheal cartilages was found to be ring‐shaped in these images (Figures 1f and 3f).

The entire cavity of the pupil was filled with an oval eyeball. The frontal bone and the suborbital arch formed the outer edges of the eyeball. There was a trabecular bony septum between the pupils of the eye. All parrots under study had a complete bony eyeball (Figures 1j and 3h). In the obtained CT images, the eye lens was not clearly detectable, and the cranial chamber (aqueous) and the caudal (vitreous) were not distinguishable. The retina was unrecognizable. Eyeball muscles, lacrimal glands and the third eyelid (nictitating membrane) had the same attenuation and could not be separated from each other. The scleral bones were found as two indistinct lines in cross‐sectional images and circular or round in sagittal images (Figures 1, 2 and 3h).

**FIGURE 4:**
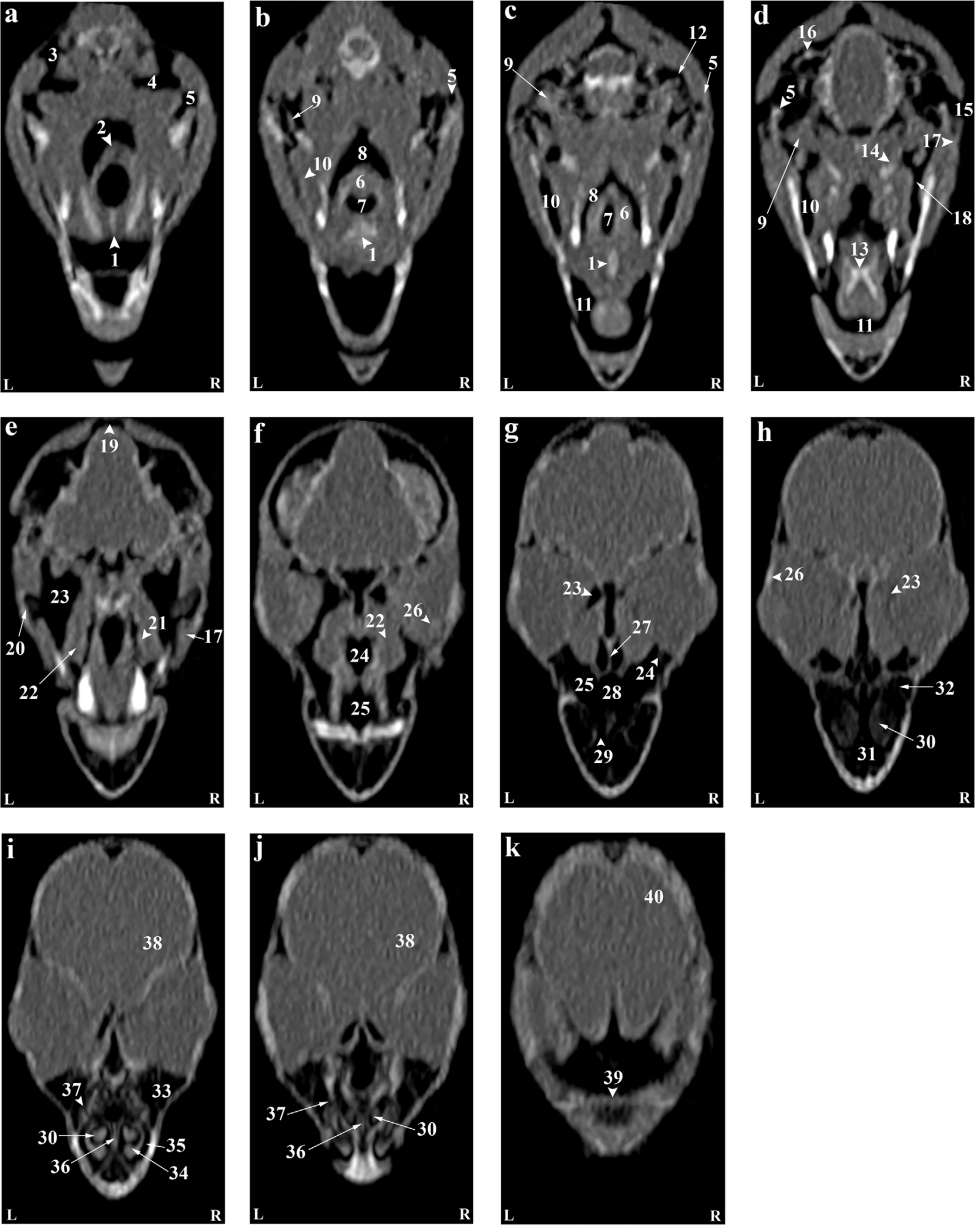
(a–k) Dorsal computed tomography reconstruction images (ventrodorsal plan) of a normal skull of the cockatiel (*Nymphicus hollandicus*) from the dorsal aspect of the skull to the larynx. (1) Basihyal, (2) arytenoid cartilage, (3) quadrature part of infraorbital sinus, (4) epithelial membrane, (5) quadrate bone, (6) larynx, (7) glottis, (8) pharynx, (9) pterygoid and quadrate muscle, (10) mandibular appendage, (11) oral cavity, (12) external acoustic meatus, (13) paraglossum, (14) pterygoid bone, (15) external ear foramen, (16) bony labyrinth, (17) jugal arch, (18) postorbital part of infraorbital sinus, (19) occipital bones (pneumonized), (20) suborbital arch, (21) palate bone, (22) ethmomandibular muscle, (23) infraorbital part of the infraorbital sinus, (24) nasopharyngeal canal, (26) scleral ossicles, (27) infraorbital septum, (28) cartilaginous part of the nasal septum, (29) palate foramen, (30) middle nasal turbinate, (31) rostral diverticulum, (32) cranial foramen of eyeball, (33) preorbital part of the infraorbital sinus, (34) rostral nasal turbinate, (35) nasal cavity, (36) bony part of nasal sinus, (37) infraorbital sinus foramen, (38) encephalon, (39) craniofacial flexion, (40) frontal bone. L, left; R, right.

The encephalon of the cockatiel (*N. hollandicus*) could be evaluated in the CT images (Figures 2c and 3i). In the cadaver samples, brain hemispheres such as the telencephalon and diencephalon, as well as the brainstem and cerebellum, were well detectable and could be distinguished from each other. However, these structures had similar attenuation in CT images, and their distinction was difficult. The findings revealed that the external acoustic meatus and the external opening of the ear of the cockatiel parakeet can be recognized in CT images (Figures 1m and 3c). Identifying the tympanic membrane in the CT images and carcasses of these birds was impossible. Hence, different parts of the middle ear were not distinguishable. Nonetheless, the presence of low‐resolution lines in the distal third of the external acoustic meatus can demonstrate parts of the middle ear, such as infraorbital (*columella*) and extracolumella cartilage. The bony labyrinth of the inner ear was well‐recognized in the cadaver samples and CT images (Figure 4).

According to our findings, the paratympanic sinus was not visible on CT images. The muscles of the head were found as hyperattenuated lines and were not highly clear. Nonetheless, relatively larger muscles, such as the quadrate, pterygoid and ethmomandibular muscles, were somehow distinguishable. Although the jaw adductor muscle is large, it was not highly detectable in CT images (Figures 1g and 3c). The infraorbital sinus was surrounded by skull bones and covering and muscular tissues and was found as a large triangular cavity that covered a large part of the head. The premaxillary bone was located in the rostral part of this sinus. In addition, the palatine and pterygoid bones were located in its inner part. Further, the quadrate, jugal arch and mandible bones were located in the lateral part. This sinus included the rostral diverticulum, transverse canal, post‐orbital, pre‐orbital, infraorbital, quadrate bones, cervicocephalic diverticulum and mandibular recess. The rostral diverticulum and the transverse canal were single, and the remaining parts were in pairs. Except for the periorbital parts, the transverse canal and the rostral diverticulum, the remaining parts of the suborbital sinus were covered by the masticatory muscle (Figures 1, 2, 3). The rostral diverticulum extended along the premaxillary bone. This diverticulum was divided into two parts by a narrow bony septum. The thickness of this septum decreased from the rostral to the caudal direction, so it completely disappeared in the middle parts of the diverticulum. The transverse channel was visible as a short and horizontal channel. The maxillary process of the palatine bone and the upper jaw‐palatine process (maxillopalatine) of the maxillary bone were located in this canal's ventral and distal parts, respectively. The transverse canal connected the periorbital region and rostral diverticulum (Figures 1, 2 and 3h). The nasopharyngeal duct divided the periorbital region into left and right parts. The jugal portion was connected dorsally with the periorbital region, ventrally with the choanal part of the palatine bone and laterally with the jugal arch. A relatively thin epithelial layer separated the periorbital from the jugal portion. These subdivisions were connected in the caudal part and placed near the infraorbital part of the infraorbital sinus. The infraorbital part was the largest part of the infraorbital sinus. It covered a large area of the ventral surface of this sinus and extended to the eyeball. This part was connected to the palatine bone and inter‐orbital septum from the medial part and to the suborbital and jugal arches from the lateral part. The infraorbital and postorbital parts were directly connected. The infraorbital and postorbital parts were the largest parts of the infraorbital sinus, respectively. The postorbital part was located in the pterygoid's lateral part, the zygomatic process's internal part and the jugal bow's posterior part, which was connected with the musculature. The masseter, pterygoid, quadrate and temporal muscles were located in the postorbital area. The caudoventral part of the postorbital was connected to the quadrate portion. The smallest part of the infraorbital sinus was related to a quadrate part, which was laterally connected with the quadrate bone. The mandibular recess and cervicocephalic diverticulum were linked to the postorbital part. The mandibular recess was visible in the inner and rostral parts of the mandibular ramus. In fact, this recess was located in the inner part of the postorbital and the ventral part of the infraorbital canal. Based on the findings, the cervicocephalic diverticulum was detectable in the skull of the cockatiel (*N. hollandicus*) and extended to the neck parts (Figures 1n and 2b).

## 4 Discussion

Based on CT and gross anatomy results, the skull of the cockatiel (*N. hollandicus*) was similar to that of other parrots, and there was no difference between the skulls of male and female parrots. The CT diagnostic method enabled the anatomical description of the skull of the cockatiel (*N. hollandicus*), which is in line with the reports of some researchers in this field (e.g., Sabat et al. 2017; Iwaniuk et al. 2004). Although the heads of these types of parrots were small, the quality and clarity required to identify the bones and tissues of the head, such as the jugal arch, palatine bone, ear ossicles and odontoglossum bones inside the mouth and different parts of the infraorbital sinus, were provided in the obtained CT images. Of course, the type of CT scanner that was employed in this study (Toshiba Multi‐slice CT scanner Asteion Premium 4, Model: TSX‐021B, Japan) played an essential role in the quality and resolution of the obtained images, and this device obtained appropriate images of the heads of these parrots. In this research, the bony trabeculae of the head of the rose‐ringed parakeet were observed by using a suitable window (WW: 2336 HU; WL: 368 HU). Parietal and temporal bones, nasal conchae, epithelial membranes, external acoustic meatus and bony labyrinth were identified as well. By performing head CT in the sagittal, transverse and dorsal planes, images of different tissues of the head were obtained, especially various parts of the infraorbital sinus, which had diagnostic value. Scanning the head in different planes solved the problem of superimposition of the images of different tissues, and each of the tissues was individually and specifically evaluated accordingly. Cubo and Casinos (2000) examined the bones of different species of birds and reported that some bones of birds contain air bubbles, which conforms to the results of our study. Based on our observations, some of the bones related to the skull of the cockatiel (*N. hollandicus*), such as the occipital, maxillary, premaxillary, mandible, palatine, pterygoid and quadrate bones, are trabecular and pneumonized and have air bubbles. In another study, Veladiano (2018) investigated the head CT of different birds and described the role of the pneumatic foramen, suborbital and paratympanic sinuses, which contradicts our observations. Based on CT images of the head of the cockatiel (*N. hollandicus*), the pneumatic foramen was undetectable, and the origin of pneumatization of the head bones could not be evaluated. Furthermore, the paratympanic sinus could not be detected in these images, which is probably due to the fusion of this sinus with middle ear tissues.

In this study, the tympanic membrane and different parts of the middle ear could not be detected in the CT images. However, the presence of low‐resolution lines in the distal third of the external acoustic meatus can demonstrate parts of the middle ear, such as columella and extracolumella cartilage. These results corroborate those of Wild's study (Wild 2015). In this study, it has been reported that the cochlea, tympanic membrane, extracolumella cartilage and columella of parrots are extremely small and thus cannot be observed in CT images. Nonetheless, it is suggested that other diagnostic imaging methods, such as micro‐CT and MRI, be used to evaluate these parts.

Some studies have indicated the presence of three conchae in the nasal cavities of birds (e.g., Langlois et al. 2020; Hanafy et al. 2021), which is consistent with our study findings. The results of the present study revealed that each nasal cavity of the cockatiel (*N. hollandicus*) consisted of a single meatus, which had caudal, middle and rostral cartilaginous conchae. Nevertheless, some other studies reported two conchae in the Congo grey parrot (*P. erithacus*) (Pohlemeyer and Kummerfeld 1989), the budgerigar (*Melopsittacus undulates*) (Orosz 2016) and the brown‐eared nightingale (*Hysipetes amaurotis*) (Yokosuka et al. 2009), or more than three conchae in the petrel (*Pagodroma* sp.) (Piro and Acosta 2019). In the nostrils of the cockatiel (*N. hollandicus*), the middle and caudal turbinates had the largest and smallest sizes, respectively, which conforms to the results of studies conducted on parrots by Hanafy (2021) and Al‐Rubaie and Kadhim (2023). In the skull of the cockatiel (*N. hollandicus*), similar to other parrots, there is a middle concha in the form of a long duct, which is located in the upper respiratory airway and originates from a basal lamella, which itself is divided into a sinus lamella and a spiral lamella. Moreover, in this type of bird, the caudal concha is small and hollow and is placed in the caudal nasal cavity. However, the results of some studies contradict our observations in this regard. For instance, Faillace et al. (2021), examining the CT results of the nasal conchae of the blue‐fronted Amazon parrot (*A. aestiva*), indicated that the middle concha is a narrow linear structure inside the rostral concha. They further found that in this type of parrot, the caudal concha can have different sizes, so that the size of this concha is large in some of these birds, while it is extremely small in others. In a study, Madkour (2019) claimed that the nasal conchae of some bird species have bone tissue in addition to cartilaginous tissue. This recent report does not match the result of our study because, according to our observations, the structure of the nasal conchae of the cockatiel (*N. hollandicus*) was purely cartilaginous, and this finding was confirmed by the attenuation of the CT images of the head. In another study, Van Zeeland (2018) investigated the upper respiratory tracts of parrots and reported that the nasopharynx is the place where the nasal cavities connect to the throat, and adenoids and most of the lymph tissues are located in this region. The reports of this study are somewhat in line with our gross anatomy results. The nasal and oral cavities were linked through the nasopharyngeal duct in the cockatiel (*N. hollandicus*). Based on CT images, the nasopharyngeal duct was rostrolaterally and caudally connected to the maxilla‐palatal process of the maxillary bone and the choanal part of the palatine bone, respectively. The caudal part of the nasopharyngeal duct was linked to the inter‐orbital septum. Unfortunately, it was impossible to find valid studies on the nasopharyngeal CT characteristics of birds and then compare their results with those of this study. It is hoped that the findings of this research pave the way for future research in this respect.

The oral cavity of the cockatiel (*N. hollandicus*) had a hyobranchial apparatus. The caudal part of this system was located in the inner part of the mandible ramus, or in other words, the cranial part of the trachea. These findings conform to the results of other studies performed on parrots. According to our observations, the base of the tongue was in close contact with the paraglossum and the cranial part of the basihyal.

The results of the present study demonstrated that the pupil of the cockatiel (*N. hollandicus*) is completely bony. In the gross anatomy studies, it was possible to determine the cranial and caudal chambers, lens and optic nerve of this bird's eye, which matches the reports of most researchers, and it seems that the eye anatomy of this type of parrot does not particularly differ from that of other birds (Moore et al. 2022). However, unlike the gross anatomical evaluations, the lens was not clearly visible, and it was impossible to distinguish the ocular chambers in the obtained CT images. The retina was also unrecognizable. The muscles of the eyeball, lacrimal glands and third eyelid (nictitating membrane) had the same attenuation and therefore could not be separated from each other. The researchers of this study could not find written and specific reports about CT scans of birds’ eyes and compare them with the results of this study. However, according to the findings of this study, it is suggested that diagnostic imaging methods such as ultrasonography, micro‐CT, MRI and other specialized eye evaluation methods be used to examine the internal tissues of the eye.

In the CT images obtained from the head of this type of parrot, the masticatory muscle could be identified due to its large size. However, other head muscles, eyeball muscles and even nerve vessels had highly close attenuation, and it was difficult to distinguish between them; thus, they did not undergo separate investigations. Different radiation factors were employed to increase the clarity and contrast of these tissues, but no suitable answer was obtained in this regard.

Based on anatomical examination of the budgerigar and Casco (African grey parrot), Smallwood (2014) reported that the cricoid cartilage of the larynx of these birds is wide and has a rostral process. In another study, Silva et al. (2020) found that the cricoid cartilage of the larynx of the cockatiel is smooth and small and has two rostral and lateral processes, which contradicts our findings. In the cockatiel (*N. hollandicus*), the larynx consisted of an annular cricoid cartilage and two pyramidal arytenoid cartilages. The cricoid cartilage was smooth and thin and had no processes. In the middle part of the cricoid cartilage, there was the procricoid cartilage, which formed the dorsocaudal part of the larynx.

In some previous studies, the anatomy of the infraorbital sinus has been described in some domestic birds such as hens, turkeys and geese (Casteleyn et al. 2018). However, there is no detailed and comprehensive report about the anatomy and CT features of the infraorbital sinus in parrots. Based on the findings of our study, the infraorbital sinus of the cockatiel (*N. hollandicus*) was surrounded by skull bones and covering and muscular tissues, and in the CT images, it was detected as a large triangular cavity that covered a large part of the head. The premaxillary bone was located in the rostral part of this sinus. In addition, the palatine and pterygoid bones were located in its inner part, and the quadrate bone, jugal arch and mandible bones were located in the lateral part. Grist (2006) conducted an anatomical study on domestic chickens and found that there were fewer infraorbital sinus chambers in the head of this type of bird. The names and characteristics of these chambers were not mentioned in this report. Eventually, it was indicated that this sinus is shorter in other birds and is limited by the infraorbital part. According to our observations, this sinus included the rostral diverticulum, transverse canal, postorbital, preorbital, infraorbital and quadrate parts, cervicocephalic diverticulum and mandibular recess in the cockatiel (*N. hollandicus*). The head and neck of this type of parrot were widely pneumatized with this sinus. No specific homologies were inferred in this regard since the analogy of the infraorbital sinus and phylogenetic evaluations between the cockatiel (*N. hollandicus*) and other parrots was impossible.

de Almeida Lima Massari et al. (2020) performed CT on the head of a macaw and concluded that the infraorbital, periorbital and rostral diverticulum of the infraorbital sinus can be easily detected, which is mainly due to the large chambers of this sinus and the absence of covering muscles in this region. It was further indicated that the postorbital, quadrate and mandibular recess parts were not detectable because they were small and superimposed by the masticatory muscle. These findings somewhat corroborate the results of our study. The findings of the present research revealed that in the cockatiel (*N. hollandicus*), except for the periorbital, transverse canal and rostral diverticulum, the remaining parts of the suborbital sinus were covered by the masticatory muscle.

In some studies, the existence of a paratracheal recess was reported in Amazon and Cockatoo (Carril et al. 2016), as well as Anodorhynchus and Ararauna macaws (Monção‐Silva et al. 2016). The results of these studies contradict the findings of our research. Based on our observations, paratracheal recess was not observed in any of the cockatiels under investigation; therefore, this feature can be mentioned in the comparative anatomy of this type of parrot.

The skull of the cockatiel (*N. hollandicus*) was relatively small, and the limit distance of its constituent bones was visible. The periorbital sinus was located in the antorbital fenestra, and the zygomatic process of the squamosal bone surrounded the postorbital sinus. In the computed tomography images, the muscles of the head of this parrot were detected as hyper‐attenuated lines and were not very clear. However, relatively larger muscles such as quadrate, the pterygoid and ethmomandibular muscles were somehow distinguishable. Although the jaw adductor muscle was large, it could not be detected in the CT images, and its boundaries was determined based on the topography of the bones in that region.

Based on the present study's findings, the columella ossicle, its external cartilage and the cochlea were not recognizable in CT images. This is mainly due to the small size of these structures. Hence, it is recommended that other diagnostic imaging methods such as micro‐CT or MRI be utilized in cases where it is intended to evaluate these structures.

The analysis of the findings and their comparison with the results of other studies revealed that the skull of the cockatiel (*N. hollandicus*) was not that much different from that of other parrots. The only morphological differences were related to some parts of the nasal cavity, the infraorbital sinus and, to some extent, the hyobranchial apparatus and nasopharyngeal duct.

## 5 Conclusion

The CT scan is one of the most appropriate and valuable diagnostic imaging methods to describe and dissect most of the hard and soft tissues of the head of the cockatiel (*N. hollandicus*). The results of this study demonstrated that CT images can be used to examine the infraorbital sinus and the turbinates, or conchae, of nasal cavities. The investigation of the tomographic features of the head of the cockatiel (*N. hollandicus*) can be useful in identifying anatomical features and evaluating its pathological cases. This study investigated the normal anatomy of the cockatiel (*N. hollandicus*) head by CT using 3D modelling. The simultaneous study of CT evaluation and anatomical examination of the head of the cockatiel represented highly correlated findings. The results of this research can be utilized as a reference and atlas for identifying anatomical characteristics, examining various species of cockatiel (*N. hollandicus*), teaching anatomy and interpreting CT scan images. Moreover, they can be used for clinical examinations and treatment of this type of parrot.

## Author Contributions

**Seyedmehran Kazemi**: resources, software, formal analysis, investigation, project administration. **Mehdi Rezaei**: conceptualization, investigation, writing–original draft, writing–review and editing, visualization, validation, methodology, data curation, supervision, resources. **Siamak Alizadeh**: conceptualization, investigation, writing–original draft, writing–review and editing, visualization, validation. **Mohammadreza Hosseinchi**: conceptualization, writing–original draft, visualization, formal analysis, resources, investigation, software, data curation.

## Conflicts of Interest

The authors declare no conflicts of interest.

## Data Availability

The data that support the findings of this study are available from the corresponding author upon reasonable request.
